# Prevalence of Hyperkalemia in a Contemporary European Cohort According to EKFC eGFR Categories

**DOI:** 10.3390/diagnostics16091309

**Published:** 2026-04-27

**Authors:** Priscila Villalvazo, Luis Miguel Molinero-Casares, Maria Dolores Sanchez-Niño, Alberto Ortiz

**Affiliations:** 1 Department of Nephrology and Hypertension, IIS-Fundacion Jimenez Diaz, Universidad Autónoma de Madrid, 28040 Madrid, Spain; prizvillalvazo@gmail.com (P.V.); mdsanchez@fjd.es (M.D.S.-N.); 2 Department of Medicine, Universidad Autónoma de Madrid, 28029 Madrid, Spain; 3 Alce Ingeniería, 28040 Madrid, Spain; molinerolm@alceingenieria.net; 4 Department of Pharmacology, Universidad Autónoma de Madrid, 28029 Madrid, Spain

**Keywords:** chronic kidney disease, hyperkalemia, hypokalemia, EKFC, glomerular filtration rate, KDIGO

## Abstract

**Background/Objectives**: Hyperkalemia is common in patients with chronic kidney disease (CKD). However, its epidemiology may be evolving due to population aging, new therapeutic developments and novel estimated glomerular filtration rate (eGFR) equations. We have re-evaluated the epidemiology of hyperkalemia in a contemporary cohort in which eGFR was assessed using the EKFC equation recommended by the European Federation of Clinical Chemistry and Laboratory Medicine (EFLM). **Methods**: We analyzed 190,579 laboratory tests with serum potassium values corresponding to individual outpatients in Primary or Specialty Care from a single laboratory in 2023, representing 42% of the catchment area population. **Results**: Hypokalemia (<3.5 mmol/L) was present in 0.3% patients, hyperkalemia (≥5.0 mmol/L) in 10.5% (11.5% of men, 9.7% of women). Hyperkalemia was mostly mild (9.4%) but was severe in 0.1% overall and in 10.5% of CKD G5. One in four patients with hyperkalemia had CKD. Hyperkalemia was more common among patients with CKD G3–G5 defined using the CKD-EPI2009 equation than defined using the EKFC equation (20.5 vs. 18.6%, *p* < 0.0001). Using EKFC, hyperkalemia prevalence increased with decreasing eGFR from G1 (6.6%) to G2 (10.8%) and, especially in CKD G3–G5 (G3 17.2% to G5 47.5%). In multivariate logistic analysis, worse renal function, worse diabetes control, older age, and surrogates for release of intracellular potassium during sample processing (red blood cell counts or size, platelet counts, elevated calcium levels) were independently associated with hyperkalemia. This multivariate model yielded an area under the curve (AUC) of the Receiver Operating Characteristic (ROC) curve for hyperkalemia of 0.678 (95% CI 0.674–0.682). Random forest also identified GFR as the most important feature associated with hyperkalemia and generally concurred with logistic analysis findings. **Conclusions**: Hyperkalemia remains common, especially in CKD G5. While hyperkalemia is mainly associated with low eGFR, sample processing should be optimized.

## 1. Introduction

Chronic kidney disease (CKD) is forecast to become the third cause of death in multiple countries by 2050 [1,2]. CKD is defined by a low kidney function or evidence of kidney injury for longer than 3 months, which are associated with adverse outcomes [3,4]. The glomerular filtration rate, usually estimated (eGFR) from serum creatinine, and albuminuria (urinary albumin-creatinine ratio, UACR) are used to both diagnose and risk-stratify CKD. The CKD-EPI2009 equation is widely used in Europe in a race-free manner to estimate GFR from serum creatinine [5,6], but the United States dropped it to implement CKD-EPI2021, a novel race-free equation [7,8]. The most recent KDIGO 2024 Clinical Practice Guideline on CKD recommends using a validated race-free equation to derive eGFR from serum filtration markers (e.g., serum creatinine) within as large as possible geographical regions and considers both CKD-EPI2021 and the European Kidney Function Consortium (EKFC) equation as validated [4]. Based on this recommendation, the European Federation of Clinical Chemistry and Laboratory Medicine (EFLM) endorsed the EKFC equation for use in Europe [9]. Compared to CKD-EPI2009 or CKD-EPI2021, EKFC has smaller bias in Europe [10], and resulted in lower eGFR values. This translated into a higher prevalence of CKD G3–G5 [11,12]. A shift in the CKD population may potentially modify the epidemiology of hyperkalemia associated with CKD and influence hyperkalemia-related nutritional advice.

Potassium disturbances may be a consequence of both CKD and of its treatment [13,14,15,16,17,18]. Tubulopathies may cause hypokalemia while a low eGFR and CKD-associated tubular dysfunction limits the ability to excrete potassium, increasing the risk of hyperkalemia [18]. Certain causes of CKD, such as diabetes mellitus are also associated with hyperkalemia, due to an impaired buffering capacity to the intracellular space and altered potassium excretion [16,17,18]. Additionally, some treatments, such as thiazide and loop diuretics or peritoneal dialysis may cause hypokalemia while renin–angiotensin–aldosterone system blockers can cause hyperkalemia, which may limit their use, resulting in impaired outcomes due to undertreatment [16,18]. Potassium disturbances adversely impact patient outcomes through multiple mechanisms. Both hypokalemia and hyperkalemia may be lethal and adversely impact quality of life via the need for additional medication, dietary changes or dyskalemia-associated symptoms [18].

Additionally, the recent availability of novel oral potassium binders (patiromer from 2017, sodium zirconium cyclosilicate, SZC, from 2018), as well as kidney protective medications, such as SGLT2 inhibitors [4,13,14], which reduce the risk of hyperkalemia [13,14,15,16,17,18] may have modified the epidemiology of hyperkalemia in CKD. However, there are few contemporary reports on the prevalence of hyperkalemia in large combined Primary and Specialty Care outpatient datasets, and most available data predate the widespread availability of the new treatments [18].

Since the epidemiology of potassium disturbances may be evolving due to population aging, new therapeutic developments and novel estimated glomerular filtration rate (eGFR) equations, the aim of the present study was to analyze the epidemiology of hypokalemia and hyperkalemia in a large contemporary dataset combining outpatient Primary and Specialty Care, defining CKD according to EKFC eGFR categories. The study was performed in Madrid, the European region with the longest life expectancy [19] which allowed us to generate data across the full adult age range spectrum. This kind of data may be useful to plan future care needs for healthcare systems and policymakers, as the global population ages [20,21].

## 2. Materials and Methods

### 2.1. Study Design and Data Processing

This observational, retrospective and cross-sectional study took advantage of a single central clinical laboratory serving both Primary and Specialist Care from Primary Care centers and a large referral hospital in an urban healthcare catchment area. Laboratory assessments from outpatients from 1 January to 31 December 2023 were included while inpatient or emergency room samples were excluded. The Madrid public healthcare system assigns catchment areas to hospitals based on home addresses and this catchment area includes 451,269 people [22]. Only one blood sample was analyzed per person and the last available assessment was chosen for this purpose. CKD was diagnosed using KDIGO eGFR and UACR thresholds [4]: eGFR ≤ 60 mL/min/1.73 m^2^ was assigned CKD G3–G5 (G3 30–59 mL/min/1.73 m^2^, G4 15–29 mL/min/1.73 m^2^, G5 < 15 mL/min/1.73 m^2^). As samples were from outpatients, who are generally stable and many of whom are assessed annually, there were concerns that requiring a second eGFR assessment separated by more than 3 months may exclude a significant number of patients that lacked two assessments in 2023. Thus, in line with other cross-sectional studies, including the meta-analysis used to derive the current thresholds to diagnose CKD [23,24,25,26], only one serum creatinine sample was used to estimate and categorize eGFR. CKD G1–G5 included patients with eGFR category G1 (≥90 mL/min/1.73 m^2^) or G2 (60–89 mL/min/1.73 m^2^,) who also had UACR ≥ 30 mg/g. However, albuminuria was only available for 33,789 patients.

Electronic health records were used to automatically and randomly assign a unique novel identifier to maintain anonymity and extract demographic characteristics, key comorbidities (hypertension, diabetes) and laboratory data. Extreme values (age < 18 or >105 years, analytical values considered to be clinically implausible, such as serum creatinine < 0.4 mg/dL) were excluded. eGFR was recalculated from serum creatinine, sex, and age using the eGFR equation currently in use (CKD-EPI2009 without race correction) as well as EKFC [4,12,27,28,29] (Appendix A). EKFC was used for the main analysis, while CKD-EPI2009 was only used when indicated, to compare with EKFC data. According to KDIGO, hypokalemia was defined as serum potassium < 3.5 mmol/L and hyperkalemia as ≥5.0 mmol/L [18]. For the main analysis, the severity of hyperkalemia was categorized as mild <5.5 mmol/L, moderate 5.5–<6.0 mmol/L, or severe ≥ 6.0 mmol/L, following the European Society of Cardiology Heart Failure guidelines [30] which are aligned with other guidelines [31,32]. The ESC guideline categorization of severity was chosen because it is less ambiguous than KDIGO: KDIGO required simultaneous knowledge of ECG changes, that were not available in our dataset of outpatient data, as serum potassium 6.0–6.4 mmol/L is considered both moderate or severe depending on the presence of electrocardiography changes [18]. To bridge the gap between KDIGO and ESC guidelines, we also report separately the serum potassium categories of 5.0 mmol/L and ≥6.5 mmol/L. Missing values are shown in Table S1.

### 2.2. Ethics

The study was approved by the Ethics Committee of the IIS-FJD UAM (Autonomous University of Madrid) (EO014-22_FJD, date 22 March 2022) and conducted in accordance with the Declaration of Helsinki (Edinburgh, 2000) and subsequent updates.

### 2.3. Statistical Analysis

Statistical analysis used R version 4.2.1. Quantitative variables were expressed as median (interquartile range, IQR) and compared using Student’s *t*-test or the non-parametric Mann–Whitney U test, depending on data distribution. Qualitative variables were expressed as frequency and percentage and compared using a Chi^2^ or Fisher’s exact test. Statistical significance was set at *p* ≤ 0.05. Logistic multivariate analysis and random forest were used to assess factors associated with hyperkalemia. For logistic multivariate analysis a correlation matrix was generated and variance inflation factor (VIF) values were assessed to exclude relevant collinearity (i.e., VIF values above 5 to 10).

## 3. Results

### 3.1. Serum Potassium Disturbances

A total of 216,637 analytical records from 2023 were assessed from 216,637 individual adult outpatients, out of a catchment area population of 451,269 people [22], i.e., in 2023, 48% of the catchment area population had blood biochemistry results. In this contemporary cohort, 190,579 patients had serum potassium values, of whom 110,906 (58%) were women. Figure S1 shows the age distribution. As compared with the city of Madrid general population, younger people were underrepresented in the dataset, which is expected, given that they are generally in better health and might not use the public healthcare system each year. However, for older people (aged >50 years) the age and sex distributions were very similar in the dataset and the general population. People aged over 100 years were also represented.

Median serum potassium levels were 4.5 (4.3–4.7) mmol/L both in men and in women. Hypokalemia was present in 659 (0.3%) patients: 254 (0.3%) men and 405 (0.4%) women. Hyperkalemia was more prevalent than hypokalemia: 19,971 (10.5%) cases. Hyperkalemia was more common in men (9160, 11.5%) than in women (10,811, 9.7%, *p* < 0.0001) (Table 1). Most cases of hyperkalemia were mild (17,937, 9.4%) but 162 (0.1%) were severe.

**Figure 1:**
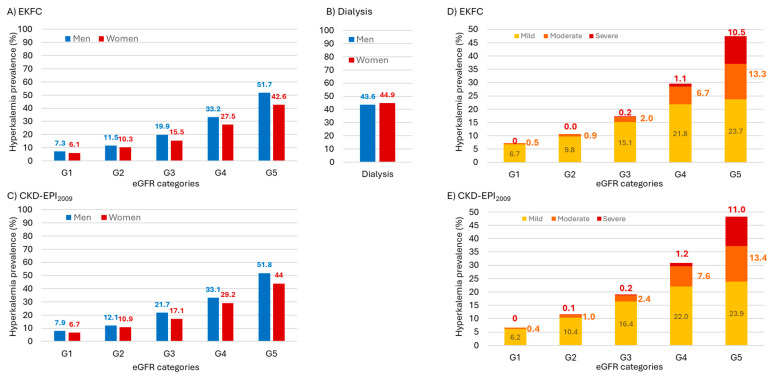
**Prevalence of hyperkalemia according to EKFC and CKD-EPI_2009_ eGFR G categories and in patients on dialysis**. (**A**) Prevalence of hyperkalemia, G categories defined based on EKFC eGFR. (**B**) Prevalence of hyperkalemia in patients on dialysis. (**C**) Prevalence of hyperkalemia, G categories defined based on CKD-EPI_2009_ eGFR. (**D**) Prevalence of mild, moderate and severe hyperkalemia, G categories defined based on EKFC eGFR. (**E**) Prevalence of mild, moderate and severe hyperkalemia, G categories defined based on CKD-EPI_2009_ eGFR. Note that in (**D**,**E**) the scale differs from (**A**–**C**).

We first assessed eGFR using the EKFC equation. The prevalence of hyperkalemia increased as eGFR decreased, ranging from 6.5% for eGFR category G1 to 47.5% in category G5 and 44% for those on dialysis (Table 2). Overall, hyperkalemia was 2-fold more prevalent in people with CKD G3–G5 than in those in the eGFR G1 and G2 categories. Additionally, it was more common in people with CKD G1–G2, as determined in the subgroup with available albuminuria testing, than in the wider eGFR G1 and G2 categories (some of whom may also have undiagnosed CKD because albuminuria values were not available). Hyperkalemia was also more prevalent in people with anemia, hypertension or diabetes. Hyperkalemia was more common in men than in women for all eGFR categories, with the difference increasing as eGFR values got lower (Figure 1A). Differences between men and women were less noticeable on dialysis (Figure 1B). Hyperkalemia was also more common in men than in women with anemia, hypertension or diabetes (Table 2).

The CKD-EPI2009 equation, currently used in most of Europe, provides eGFR values that are higher than those obtained with EKFC across most of the age range [11,12]. Despite the different prevalence of CKD G3–G5 when assessed by EKFC eGFR or CKD-EPI2009 eGFR, median serum potassium and the interquartile range largely overlapped for patients with CKD G3–G5 according to both eGFR equations (Table S2). However, patients having CKD according to EKFC but not according to CKD-EPI2009, had lower median serum potassium levels.

Hyperkalemia was more common among patients with CKD G3–G5 defined using the CKD-EPI2009 equation (Figure 1C) than when the EKFC equation was used (Figure 1A): 20.5 vs. 18.6%, *p* < 0.0001. Indeed, patients with CKD G3–G5 according to EKFC but not CKD-EPI2009 had a lower hyperkalemia prevalence (13.8%). Differences were largely explained by a higher prevalence of hyperkalemia in CKD G3 both in men (prevalence 9.0% higher for CKD-EPI2009 G3 than for CKD G3 diagnosed using EKFC) and women (10.3% higher) (Figure 1A,C). Differences in prevalence between EKFC- and CKD-EPI2009-defined G categories were larger for mild hyperkalemia in CKD G3 (prevalence 15.1 vs. 16.4%), moderate hyperkalemia in G4 (prevalence 6.7 vs. 7.6%) and severe hyperkalemia in G5 (prevalence 10.5 and 11%) (Figure 1D,E).

Severe hyperkalemia was present in 0.4% of patients with CKD G3–G5 EKFC and in 0.5% of patients with CKD G3–G5 CKD-EPI2009.

### 3.2. Characteristics of Patients with Hyperkalemia

Table 3 summarizes characteristics of patients with and without hyperkalemia. Patients with hyperkalemia were older (63 [IQR 51–75] vs. 56 [42–70] years), more frequently men and had more comorbidities. They also had a higher prevalence of diabetes (16.3% vs. 9.4%), the main cause of CKD, and higher levels of diabetes-associated parameters (glucose, HbA1C), as well as higher prevalence of CKD G3–G5 (25.6% vs. 13.1%) and CKD complications such as anemia and hypertension, and more severe changes in CKD-associated parameters (lower eGFR, higher serum creatinine, urea, uric acid and albuminuria values). Patients with hyperkalemia also had higher values of some intracellular enzymes (LDH, alkaline phosphatase, GGT), mineral metabolism parameters (calcium, phosphate. PTH, 25OH vitamin D) and evidence of inflammation (CRP, leukocytes, neutrophils, ferritin) and lower values of total CO_2_. Interestingly, platelet numbers were higher and red blood cells were larger in patients with hyperkalemia, the latter not explained by lower folic acid or vitamin B12 values. Indeed, there was a statistically significant albeit weak correlation between serum potassium and calcium values (r 0.146, *p* < 0.001).

### 3.3. Diabetes and Hyperkalemia

Hyperkalemia was 1.74-fold more common among people with diabetes than among those without diabetes (Table S3) and the difference increased with hyperkalemia severity, reaching 4.5-fold for severe hyperkalemia. Assessment of G categories disclosed that people with diabetes developed hyperkalemia with better preserved kidney function and differences were no longer apparent between people with and without diabetes in presence of more advanced CKD (Tables S4 and S5).

### 3.4. Factors Associated with Hyperkalemia: Beware of Sample Processing

**Figure 2:**
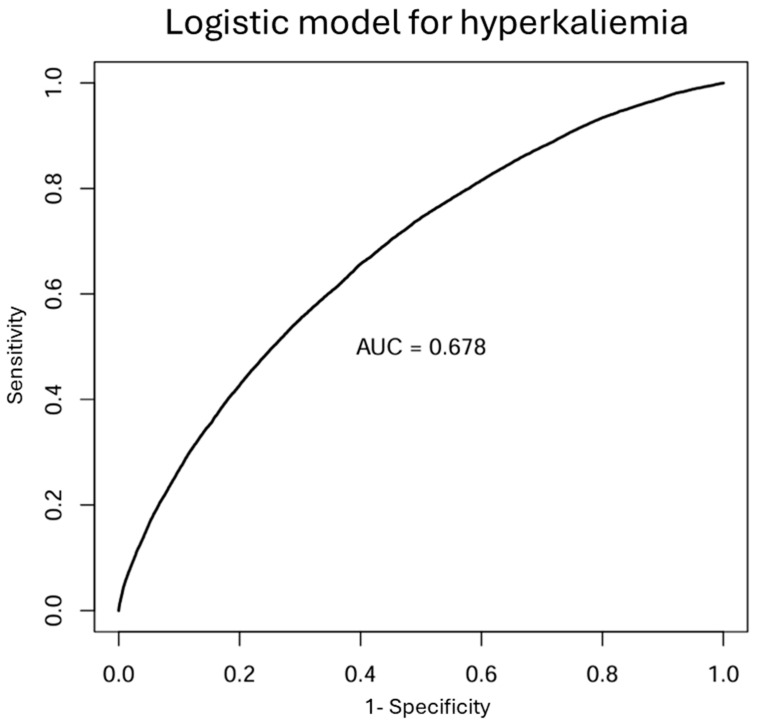
AUC for the ROC curve for the logistic model for hyperkalemia.

Logistic multivariate analysis disclosed that older age, lower kidney function (lower eGFR estimated by EKFC, higher urea and phosphate), higher number and/or size of blood cells (estimated from higher hemoglobin, mean red blood cell corpuscular volume, platelet numbers), diabetes/poorer diabetes control (higher HbA1C), and higher calcium levels were independently associated with hyperkalemia (Table 4). Table S6 shows the correlation matrix and VIF values. Model accuracy was assessed by a Receiver Operating Characteristic (ROC) curve (Figure 2). The area under the curve (AUC) was 0.678 (95% CI: 0.674–0.682), indicating a modest discrimination capacity in predicting hyperkalemia.

**Figure 3:**
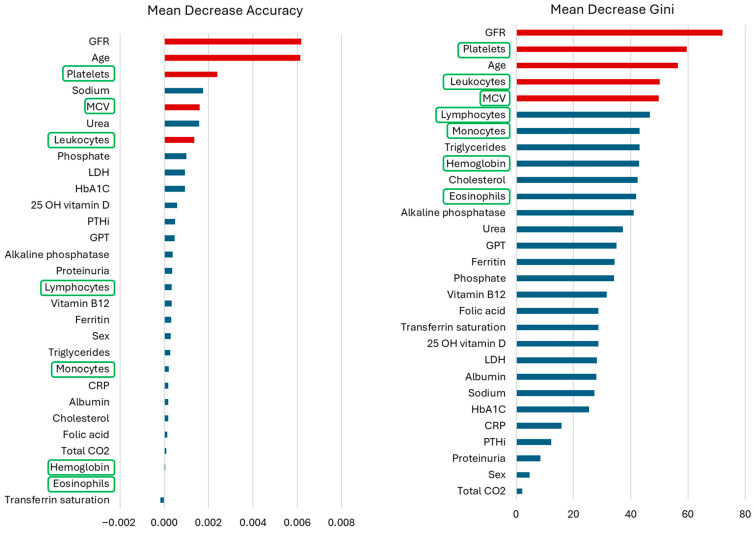
**Multivariate random forest model for hyperkalemia.** (**left**) Mean decrease accuracy. Variables ranked by descending importance of how much accuracy (successful classification) the model loses by excluding each variable. (**right**) Mean decrease Gini. It measures the contribution of each variable to the homogeneity of nodes and leaves from the random forest. Higher mean decrease accuracy or mean decrease Gini scores imply higher importance of the variable in the model. The top five features common to both assessments are color-coded red. Green color coding rectangles indicate variables that are related to higher numbers or size of blood cells.

A multivariate random forest model concurred with the main findings of the logistic analysis (Figure 3). The most important feature associated with hyperkalemia as assessed by impurity-based (Gini) importance or mean decreased accuracy was eGFR. As for logistic analysis, other factors associated with hyperkalemia included older age and parameters associated with poorer kidney function and more or larger circulating blood cells.

## 4. Discussion

The main findings are that the implementation of EKFC to assess eGFR may lead to an apparent decrease in the prevalence of hyperkalemia in people with CKD and that there is evidence that potassium release may be contributing to hyperkalemia values. This is likely unavoidable for a centralized laboratory serving multiple peripheral Primary Care centers where blood is sampled. However, it is worth acknowledging this fact as it may influence clinical decision-making. On a wider frame, hyperkalemia remains common in the era of novel potassium binders and medications, such as SGLT2 inhibitors, that protect from hyperkalemia. Of more immediate concern, severe hyperkalemia was still observed in around 10% of people with kidney failure (GFR category G5, eGFR < 15 mL/min/1.73 m^2^).

KDIGO recently defined hypokalemia as a potassium concentration < 3.5 mmol/L and reported prevalence of 1% to 3% in the general and CKD populations [18]. No specific prevalence was given for outpatient hyperkalemia, likely representing the diverse datasets available, using different definitions and settings. In an individual-level data meta-analysis of 27 international cohorts (general population, high cardiovascular risk, or CKD) in the CKD Prognosis Consortium before 2018, the prevalence of serum potassium > 5.0 mmol/L, and >5.5 mmol/L, was 3.31% [95% confidence interval (CI) 3.28–3.34%] and 0.49% (95% CI 0.48–0.50%) in individuals in the general population/high CV risk cohorts, while hypokalemia (<3.5 mmol/L) was 1.91% (95% CI 1.89–1.94%) [33]. Corresponding figures for CKD cohorts were 17.94% (95% CI 17.58–18.31%) and 4.23% (95% CI 4.03–4.42%) for hyperkalemia categories and 2.03% (95% CI 1.90–2.17%) for hypokalemia. However, wide heterogeneity has been observed according to local practice. As an example, a Chronic Kidney Disease Outcomes and Practice Patterns Study (CKDopps) analysis of 5870 patients with CKD from Brazil, Germany, France, and the United States (US) prior to 2018 found different ratios of G3:G4:G5 and of uptake of renal–angiotensin system blockers between countries [34]. Consequently, the prevalence of hyperkalemia > 5.0 mmol/L ranged from 20% to 35% for G3–G5, 13% to 25% for G3, 22% to 38% for G4 and 27% to 41% for G5.

Given the potential variability by country, we focused on Spanish data. In Spain, the NEFRONA multicenter cohort reported on 483 controls without CKD and 2408 CKD patients without prior cardiovascular disease followed by Nephrology [35]. Data were collected in 2009–2011. Median serum potassium in patients with CKD [4.8 (4.4–5.2)] was higher than in the contemporary cohort described here [4.6 (4.3–4.9)], while it was similar for non-CKD participants [4.5 (4.2–4.7) and 4.5 (4.3–4.7), respectively]. The prevalence of hypokalemia was 0.8% among controls and 2.4% among patients with CKD G3–G5 in NEFRONA, values higher than in the contemporary cohort. The prevalence of hyperkalemia > 5.0 mmol/L was 8.9% among controls (none with severe hyperkalemia) and 33.6% among patients with CKD G3–G5 (3.2% with severe hyperkalemia) in NEFRONA. While the prevalence of hyperkalemia in the non-CKD population was similar to the present study, hyperkalemia among patients with CKD was less common in the contemporary cohort. By GFR G category, NEFRONA reported 22% prevalence of hyperkalemia in G3 and 41% for G4–G5, as compared with 17.2% and 32.6%, respectively, for the contemporary cohort. The contemporary cohort appears to have a consistently lower prevalence of severe hyperkalemia at 0.4–0.5% in CKD G3–G5 as compared with 0.8–2.1% in the NEFRONA cohort and 1 to 3% in the CKDopps multinational study [34,35]. This may potentially be the result of therapeutic innovation. However, no definitive conclusions may be drawn on evolving epidemiology dynamics in the absence of direct comparisons with the same cohort in the past. In this regard, the contemporary cohort was drawn from all outpatient laboratory results, both from Primary Care and from Specialty Care, and was not limited to Nephrology, and thus is more representative than prior data focused on patients treated by Nephrology [34,35].

Lower GFR and older age are the main risk factors for hyperkalemia and the prevalence of both is increasing as populations age. The choice of eGFR equation may influence the epidemiology of hyperkalemia in CKD. Indeed, changes in the equation used to estimate GFR from serum creatinine may be associated with changes in the prevalence of hyperkalemia among patients with CKD. Specifically, the prevalence of hyperkalemia in CKD G3–G5 was 10% higher when CKD was defined using the CKD-EPI2009 equation (widely used in Europe in 2025) than when using the EKFC equation proposed by the EFLM (9). EKFC results in lower eGFR values than CKD-EPI2009, thus increasing CKD prevalence by incorporating patients that have milder CKD than those identified by CKD-EPI2009, including better preservation of potassium homeostasis. This will make comparisons across time and geography more challenging. As an example, the US has moved away from CKD-EPI2009 to CKD-EPI2021, a race-free equation that overestimates eGFR in white Americans as compared to CKD-EPI2009 and underestimates eGFR in black Americans as compared to race-containing CKD-EPI2009 [7,8]. A recent move in Europe towards adopting the European EKFC equation [9] may result in an increased prevalence of CKD [11,12], associated with milder hyperkalemia prevalence, as suggested by the present report. Comparative studies in the future should strive to define CKD using the same equation in all datasets.

The logistic multivariate analysis provides interesting insights into factors associated with hyperkalemia. Interestingly, the main categories of variables independently associated with hyperkalemia included those representing CKD and its causes (such as diabetes) but also increased numbers and/or size of blood cells and higher serum calcium levels. The independent association of multiple variables related to CKD may represent the suboptimal nature of eGFR as a measure of kidney function [36,37,38]. By contrast, the association with measures related to blood cell number and size and calcium raises the spectrum of potassium release from the intracellular to the extracellular space during blood processing to obtain serum [39].

On one hand, eGFR does not appear to capture the full impact of CKD on hyperkalemia, given the independent association with urea and phosphate. Both serum urea and phosphate increase when eGFR decreases but have additional influences (e.g., diet and metabolic bone disorders) and are not so dependent on muscle mass variability as eGFR. The contribution of other comorbidities appears to be represented by HbA1C (diabetes) and older age. The association with higher red blood cell number and volume and higher platelet number may be related to extracorporeal release of potassium from inside circulating blood cells after sample extraction, as is the association of higher calcium levels with hyperkalemia. Potassium can be measured in plasma or serum. Plasma represents the real extracellular potassium concentration in vivo. Clotting to generate serum may release potassium from platelets and if the sample is not processed immediately, further potassium may be released from erythrocytes and other blood cells [40]. While under optimal conditions, the difference between plasma and serum values is thought to be minimal, for individual patients, it may be larger. The Nordic Reference Interval Project (NORIP) reported a normal reference range for serum potassium of 3.6–4.6 mmol/L, similar to plasma potassium (3.5–4.4 mmol/L) [41]. However, the overall minimal 0.1 to 0.2 mmol/L difference may already push some patients over the 5.0 mmol/L threshold. Additionally, individual differences of up to 0.45 mmol/L were observed between serum and plasma potassium, especially among people with higher potassium levels, even within the normal range [40]. As NORIP was performed under ideal conditions, variability is expected to be higher under real-world conditions, especially if sampling and analysis are not located in the same physical space, as is the case for a central laboratory testing samples drawn from multiple Primary Care centers, as represented by the present manuscript. Our results are consistent with the influence of sample processing on the prevalence of hyperkalemia. Higher platelet, leucocyte and erythrocyte numbers or size may be associated with a larger amount of intracellular potassium in the blood sample (and of intracellular calcium or phosphate) that may be released from blood cells. Its impact may only become apparent when assessing a large database and using a non-biased approach to data analysis, as we did. In this regard, the association with higher calcium levels might also be related to release of intracellular calcium, mainly from platelets but potentially also due to minor hemolysis [42] and phosphate which is released from erythrocytes upon hemolysis, especially if blood is not centrifuged within 2 h [43]. Increased serum potassium had been previously reported in people treated with erythropoietin or hypoxia-inducible factor (HIF) stabilizers to treat anemia in CKD, two treatments that increase red blood cell numbers [44,45,46]. While this had been attributed to lower dialysis potassium clearance due to higher hematocrit in patients on hemodialysis, similar changes have been observed in non-dialysis patients [44]. The impact of treatments for anemia on serum potassium has not been consistent, raising the possibility that it may have been facilitated by the release of potassium from a higher number of erythrocytes during sample processing. While phosphate accumulates in CKD and its concentration would be expected to move in the same direction as potassium when eGFR decreases, CKD is associated with lower serum calcium levels. Thus, the association between higher serum calcium and hyperkalemia cannot be explained by worse kidney function. In this regard, 89% of hyperkalemia cases were mild and clinical laboratories, such as the local laboratory in the present study reports reference values for potassium up to 5.1 mmol/L (i.e., hyperkalemia would be ≥5.2 mmol/L), which is in contrast to the KDIGO clinical guideline definition of hyperkalemia (≥5.0 mmol/L). However, the ≈10% prevalence of severe hyperkalemia in CKD G5 is concerning and offers margin for improvement by optimizing therapy, including the prescription of potassium binders.

Being a study performed in Europe, the CKD-EPI2021 equation [7] was not tested, despite being more recent than CKD-EPI2009. CKD-EPI2021 was designed to address a United States racial issue [47,48,49,50] that does not apply to Europe, where CKD-EPI2009 is applied without race correction in many countries, including Spain, as race is not recognized as a valid parameter to categorize the population and is not available in administrative health records. CKD-EPI2021 overestimates mGFR by around 6 mL/min/1.73 m2 in white Europeans and does not improve over CKD-EPI2009 for other races [10,51]. This may delay kidney care. For this reason, both the European Renal Association (ERA) and the European Federation of Clinical Chemistry and Laboratory Medicine (EFLM) have argued against its use in European populations [8,9]. Both organizations are aligned with the KDIGO 2024 recommendation of using the same validated eGFR equation (e.g., EKFC, CKD-EPI2009 or CKD-EPI2021) in as wide as possible geographical regions [4] while avoiding adopting an equation that may adversely impact kidney health.

While KDIGO clearly differentiates between eGFR categories G1–G2 and CKD G1–G2 [3,4], both concepts can be mixed-up in the literature (e.g., [52]). CKD G1–G2 is diagnosed when people with eGFR category G1–G2 have, additional, evidence of CKD, such as albuminuria A1--A2, polycystic kidneys or other. Since albuminuria is not routinely tested, even in populations where albuminuria testing is recommended [53,54], CKD G1–G2 is underdiagnosed. We have now assessed CKD G1–G2 in the subpopulation where albuminuria testing was available. The prevalence of hyperkalemia in this CKD subpopulation was higher than in the wider eGFR G1–G2 category. Both more advanced kidney injury despite preserved eGFR and medications used to lower albuminuria may contribute to this observation.

Several limitations should be acknowledged. We lacked information on medication taken by participants. Thus, drug prescription, compliance, efficacy or effectiveness were not assessed. We just provided a current image of the epidemiology of hyperkalemia in an era when these drugs are available, without analyzing nor discussing whether their use is implemented. There was no information on some key comorbidities such as heart failure and cardiovascular disease because of a lack of access to Primary Care diagnosis EHR. The study population did not represent the full local population, and was skewed towards more elderly people, i.e., towards those seeking healthcare. Blood sampling took place both at the central laboratory and also at peripheral Primary Care centers, from where it was transported to the central laboratory. This may have facilitated the release of potassium from blood cells. Since finerenone was commercialized in Spain in June 2024, the present study has not captured any potential impact of this medication, which may increase hyperkalemia risk [55]. Smoking data was not available. Smoking is a risk factor for hyperkalemia in men but not in women with CKD [34]. Finally, given the outpatient nature of the analytical dataset, that deliberately excluded acute events as represented by emergency room visits and hospitalization episodes, information on acute events, such as gastrointestinal bleeding was not available. While the laboratory provides information on the degree of hemolysis, these data were not extracted. Unfortunately, information on time delay from sampling to analysis, transport times, or time-to-centrifugation cannot be obtained retrospectively. We do know that it is expected to be heterogeneous, given that blood could be drawn at different primary healthcare centers, during a period of several hours in the morning before being transported to the central lab while other samples were obtained at the hospital blood sampling facilities for outpatients. While not having that information is a limitation, the study is representative of routine clinical care, which is a strength.

The study also had additional strengths. It had a large database representative of the population seeking healthcare, both at Primary Care and Specialty Care levels and Specialty Care included all medical and surgical specialties. Indeed, it represented a significant proportion of the full population of the healthcare catchment area. Furthermore, all samples were processed in the same clinical laboratory and sampling at different facilities representing real-world clinical practice. Older age was well represented, with participants over the age of 100 years. Thus, this study performed in the European region with the longest life expectancy may be informative for future developments in other regions.

## 5. Conclusions

In conclusion, hyperkalemia remains a common problem, despite recent advances in prevention and treatment. In this regard, two key risk factors, CKD and older age, are becoming more common. CKD is associated with hyperkalemia even in people with relatively preserved EGFR (i.e., those with CKD G1–G2). Changes in eGFR equations may influence the epidemiology of hyperkalemia in CKD. While hyperkalemia is mainly associated with low eGFR, sample processing may be optimized, as suggested by the association of hyperkalemia with analytical parameters related to a higher number of circulating blood cells or a larger size of blood cells, as well as the association with serum calcium levels. In this regard, clinical laboratories account for unavoidable sample processing issues derived from sampling in multiple different geographical locations for a single centralized laboratory by providing wider reference values for serum potassium, but clinicians follow clinical guideline definitions of hyperkalemia. Communication between clinical laboratories and clinicians is key for optimal decision-making.

## Figures and Tables

**Table 1:** Serum potassium disturbances.

	Total, *n* = 190,732	Men, *n* = 79,826	Women, *n* = 110,906
Hypokalemia, *n* (%)	659 (0.3%)	254 (0.3%)	405 (0.4%)
Hyperkalemia all, *n* (%)	19,971 (10.5%)	9160 (11.5%)	10,811 (9.7%)
5.0 mmol/L *	4869 (2.5%)	2140 (2.7%)	2729 (2.5%
>5.0–<5.2 mmol/L *	4815 (2.4%)	2189 (2.7%)	2626 (2.4%)
Mild (≥5.0–<5.5 mmol/L)	17,937 (9.4%)	8188 (10.3%)	9749 (8.8%)
Moderate (5.5–<6.0 mmol/L)	1872 (1.0%)	896 (1.1%)	976 (0.9%)
Severe (≥6.0 mmol/L)	162 (0.1%)	76 (0.1%)	86 (0.1%)
≥6.5 mmol/L	31 (0.02%)	16 (0.02%)	15 (0.01%)

* Within the reference range provided by the laboratory.

**Table 2:** **Prevalence of hyperkalemia by sex.** Percentages refer to the categories shown in the first column.

Variable	All	Men	Women
eGFR categories (EKFC)			
G1, *n* (%)	4353 (6.6%)	1978 (7.3%)	2375 (6.1%)
G2, *n* (%)	10,491 (10.8%)	4843 (11.5%)	5648 (10.3%)
G3, *n* (%)	4316 (17.2%)	1966 (19.9%)	2350 (15.5%)
G4, *n* (%)	598 (29.5%)	253 (33.2%)	345 (27.5%)
G5, *n* (%)	203 (47.5%)	119 (51.7%)	84 (42.6%)
Dialysis, *n* (%)	159 (44%)	102 (43.6%)	57 (44.9%)
CKD G3–G5, *n* (%) *			
Yes	5117 (18.6%)	2338 (21.5%)	2779 (16.7%)
No	14,844 (9.1%)	6821 (9.9%)	8023 (8.5%)
CKD G1–G5, *n* (%) *			
Yes	5492 (18.0%)	2561 (20.3%)	2931 (16.4%)
No	14,469 (9.0%)	6598 (9.8%)	7871 (8.5%)
CKD G1–G2, *n* (%) **			
Yes	375 (12.1%)	223 (12.7%)	152 (11.4%)
Anemia, *n* (%) *			
Yes	2072 (15.2%)	9028 (19.8%)	1048 (12.3%)
No	17,564 (10.1%)	8004 (10.9%)	9560 (9.5%)
Hypertension, *n* (%) *			
Yes	6482 (13.2%)	3302 (14.5%)	3180 (12.2%)
No	11,675 (9.5%)	4988 (10.4%)	6687 (8.9%)
Diabetes, *n* (%) *			
Yes	3262 (16.9%)	1877 (18.2%)	1385 (15.5%)
No	14,955 (9.7%)	6446 (10.6%)	8509 (9.2%)

* *p* < 0.0001 vs. not having this condition for all, men and women. ** The percentage refers to the subpopulation with UACR data.

**Table 3:** Characteristics of patients with and without hyperkalemia.

Variable	Hyperkalemia (*n* = 19,961)	No Hyperkalemia (*n* = 170,618)	*p* Value
Age (years)	63 (51–75)	56 (42–70)	<0.0001
Female, *n* (%)	10,811 (54.2%)	100,095 (58.7%)	<0.0001
Hypertension (%)	6482 (32.5%)	42,456 (24.9%)	<0.0001
Diabetes	3262 (16.3%)	16,004 (9.4%)	<0.0001
Anemia	2072 (10.4)	11,589 (6.8%)	<0.0001
CKD G3–G5 according to EKFC, *n* (%)	5117 (25.6%)	22,367 (13.1%)	<0.0001
Dialysis	159 (0.8%)	202 (0.1%)	<0.0001
Creatinine (mg/dL)	0.9 (0.7–1.0)	0.8 (0.7–0.9)	<0.0001
eGFR CKD-EPI2009 (ml/min/1.73 m^2^)	85.1 (74.8–95.9)	90.4 (79.2–102.3)	<0.0001
eGFR EKFC (ml/min/1.73 m^2^)	75.0 (59.5–88.1)	83.1 (69.8–96.3)	<0.0001
UACR (mg/g)	12.6 (5.4–44.8)	8.0 (4.3–21.1)	<0.0001
Urea (mg/dL)	39 (32–51)	35 (29–43)	<0.0001
Uric acid (mg/dL)	5.1 (4.2–6.1)	4.8 (4–5.8)	<0.0001
Glucose (mg/dL)	95 (87–107)	92 (85–102)	<0.0001
HbA1c (%)	5.6 (5.3–6.2)	5.4 (5.1–5.8)	<0.0001
Cholesterol total (mg/dL)	188 (160–215)	187 (162–213)	0.58
Triglycerides (mg/dL)	95 (72–131)	93 (70–130)	0.17
GOT (UI/L)	19 (16–24)	19 (16–24)	0.063
GPT (UI/L)	17 (13–24)	17 (13–24)	0.49
Alkaline Phosphatase (UI/L)	78 (64–95)	74 (61–90)	<0.0001
GGT (UI/L)	19 (13–30)	17 (12–27)	<0.0001
LDH (UI/L)	181 (161–206)	176 (157–199)	<0.0001
Totals Proteins (g/dL)	7.0 (6.7–7.3)	7.0 (6.7–7.2)	0.88
Albumin (g/dL) *	4.5 (4.3–4.7)	4.5 (4.3–4.7)	<0.0001
Total CO_2_ (mmol/L)	24 (22–26)	25 (23–27)	<0.0001
Na (mmol/L) **	141 (140–143)	141 (140–143)	0.0009
K (mmol/L)	5.1 (5.0–5.3)	4.4 (4.2–4.9)	<0.0001
Cl (mg/dL)	104 (103–106)	104 (103–106)	0.24
Ca (mg/dL)	9.6 (9.4–9.9)	9.5 (9.2–9.8)	<0.0001
P (mg/dL)	3.5 (3.1–3.9)	3.4 (3.1–3.8)	<0.0001
PTH, intact (pg/mL)	52.1 (38.1–74.5)	46.5 (35.1–62.2)	<0.0001
25-OH vitamin D (ng/mL)	26.6 (19.5–35.2)	25.9 (19.1–34.1)	<0.0001
CRP (mg/L)	0.18 (0.08–0.45)	0.16 (0.07–0.38)	<0.0001
Vitamin B12 (pg/mL)	417 (318–544)	414 (320–537)	0.0537
Folic acid (ng/mL)	6.8 (4.6–10.3)	6.6 (4.5–10)	<0.0010
Hb (g/dL)	14.3 (13.3–15.4)	14.3 (13.3–15.3)	0.14
MCV (fL)	91.4 (88.3–94.6)	90.5 (87.5–93.5)	<0.0001
Leucocytes (10^3^/µL)	6.9 (5.7–8.3)	6.6 (5.5–7.9)	<0.0001
Platelets (10^3^/µL)	260 (218–309)	251 (212–295)	<0.0001
Lymphocytes (10^3^/µL)	33.1 (26.9–39.3)	34.3 (28.2–40.4)	<0.0001
Monocytes (10^3^/µL)	8.4 (7.3–9.8)	8.3 (7.2–9.7)	<0.0001
Neutrophils (10^3^/µL)	54.3 (47.8–60.9)	53.3 (46.9–59.7)	<0.0001
Eosinophils (10^3^/µL)	2.6 (1.7–3.8)	2.5 (1.6–3.7)	<0.0001
Ferritin (µL/L)	112.5 (56–209)	103 (50–196)	<0.0001
Transferrin saturation (%) *	26 (19–33)	26 (20–34)	<0.0001

* Slightly higher values in non-hyperkalemia. ** Slightly higher values in hyperkalemia.

**Table 4:** Logistic multivariate model for independent risk factors associated with hyperkalemia.

Variable	OR (95% CI)	*p* Value
eGFR	0.74 (0.72–0.75)	<0.0001
Calcium	1.31 (1.29–1.33)	<0.0001
Platelets	1.27 (1.25–1.29)	<0.0001
Phosphate	1.13 (1.11–1.15)	<0.0001
MCV	1.12 (1.11–1.13)	<0.0001
Age	1.12 (1.09–1.15)	<0.0001
HbA1C	1.11 (1.10–1.13)	<0.0001
Urea	1.10 (1.09–1.11)	<0.0001
Hemoglobin	1.09 (1.08–1.11)	<0.0001

## Data Availability

The data presented in this study are available on request from the corresponding author due ethical reasons.

## Supplementary Materials

The following supporting information can be downloaded at: https://www.mdpi.com/article/10.3390/diagnostics16091309/s1, Figure S1: Age distribution. (A) Study population. (B) City of Madrid in 2023, according to the Instituto Nacional de Estadística (https://www.ine.es/jaxiT3/Datos.htm?t=68535#_tabs-tabla; last accessed 21 August 2025). Table S1: Missing values. Table S2: Serum potassium levels and prevalence of hyperkalemia in patients with CKD G3–G5 as categorized according to the serum creatinine-based EKFC and CKD-EPI2009 equations. Table S3. Prevalence of hyperkaliemia according to diabetes status. Table S4. Prevalence of hyperkaliemia in people without diabetes according to eGFR G category. Table S5. Prevalence of hyperkaliemia in people with diabetes according to eGFR G category. Table S6. Correlation matrix for predictive variables in the logistic multivariate model and VIF values.

## Appendix A. Supplementary Methods

### GFR Equations

Both CKD-EPI2009 and EKFC are serum creatinine-based equations that have been externally validated and are recommended by the latest KDIGO CKD guidelines, which extensively discuss both. A key difference is the inclusion in EKFC of a Q value, which should be assessed for target population and consists of the normal level of creatinine in that region. Additionally, the specific EKFC equation differs for people older and younger than 40 years. Consequently, eGFR in healthy populations is stable from age 18 to 40 years as assessed by EKFC but continually decreases when assessed by CKD-EPI2009.

The equations were applied as follows:

**Table A1:** eGFR equations.

	Age	Sex	Serum Creatinine (mg/dL)	Equation
CKD-EPI_2009_	≥18	Female	SCr ≤ 0.7	144 × (SCr/0.7)^−0.329^ × (0.993)^Age^
			SCr > 0.7	144 × (SCr/0.7)^−1.209^ × (0.993)^Age^
		Male	SCr ≤ 0.9	141 × (SCr/0.9)^−0.411^ × (0.993)^Age^
			SCr > 0.9	141 × (SCr/0.9)^−1.209^ × (0.993)^Age^
EKFC	18–40	Female	SCr/Q < 1.0	107.3 × (SCr/Q)^−0.322^
			SCr/Q ≥ 1.0	107.3 × (SCr/Q)^−1.132^
		Male	SCr/Q < 1.0	107.3 × (SCr/Q)^−0.322^
			SCr/Q ≥ 1.0	107.3 × (SCr/Q)^−1.132^
	>40	Female	SCr/Q < 1.0	107.3 × (SCr/Q)^−0.322^ × 0.990^(Age−40)^
			SCr/Q ≥ 1.0	107.3 × (SCr/Q)^−1.132^ × 0.990^(Age−40)^
		Male	SCr/Q < 1.0	107.3 × (SCr/Q)^−0.322^ × 0.990^(Age−40)^
			SCr/Q ≥ 1.0	107.3 × (SCr/Q)^−1.132^ × 0.990^(Age−40)^
