# Supplementary material for: Prevalence of Hyperkalemia in a Contemporary European Cohort According to EKFC eGFR Categories
Source: Diagnostics (Basel). 2026 Apr 27;16(9):1309. doi: 10.3390/diagnostics16091309 (PMC13163007; doi:10.3390/diagnostics16091309)
Supplement: Supplementary file 1 [file diagnostics-16-01309-s001.zip › diagnostics-4205253-supplementary.pdf]

diagnostics

MDPI

# Prevalence of hyperkalemia in a contemporary European cohort according to EKFC eGFR categories

**Priscila Villalvazo[1,2], Luis Miguel Molinero-Casares[3], Maria Dolores Sanchez-Niño[1,2], Alberto Ortiz[1,2] ***

**Supplementary Materials:**

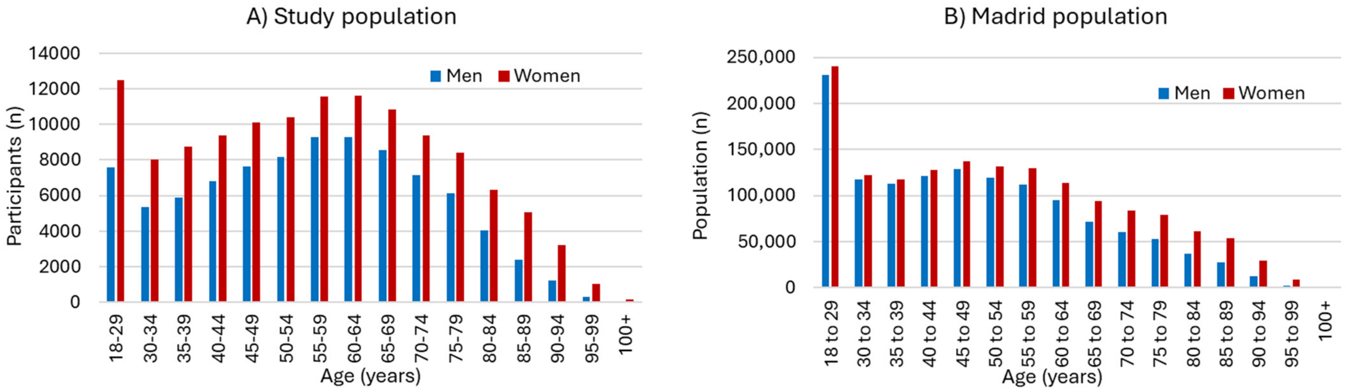


Figure S1: Age distribution. A) Study population. B) City of Madrid in 2023, according to the Instituto Nacional de Estadística (https://www.ine.es/jaxiT3/Datos.htm?t=68535#_tabs-tabla; last accessed August 21, 2025).

Table S1: Missing values

**Table S1. Missing values**

| Variable | Missing (n) |
|---|---|
| LDL cholesterol | 190138 |
| Total $CO_2$ | 184777 |
| PTHi | 160933 |
| CRP | 138929 |
| Chloride | 115732 |
| HbA1C | 110972 |
| GOT | 100354 |
| LDH | 97908 |
| 25 OH vitamin D | 95494 |
| Total protein | 94886 |
| Folic acid | 88547 |
| Urea | 86543 |
| Vitamin B12 | 77598 |
| Transferrin saturation | 67118 |
| Ferritin | 57433 |
| HDL cholesterol | 40689 |
| Phosphate | 33844 |
| Alkaline phosphatase | 27979 |
| Total bilirubin | 27718 |
| Uric acid | 25112 |
| Calcium | 24905 |
| Albumin | 21545 |
| GGT | 19474 |
| Triglycerides | 17894 |
| Cholesterol | 17483 |
| GPT | 14745 |
| Platelets | 3601 |
| Hemoglobin | 3263 |
| Neutrophils | 3245 |
| Monocytes | 3244 |
| Lymphocytes | 3241 |
| Eosinophils | 3241 |
| Leukocytes | 3235 |
| MCV | 3235 |
| Glucose | 782 |
| Creatinine | 153 |
| Sodium | 133 |

Table S2: Serum potassium levels and prevalence of hyperkalemia in patients with CKD G3-G5 as categorized according to the serum creatinine-based EKFC and CKD-EPI2009 equations

| | **CKD G3-G5 (EKFC)** | **CKD G3-G5 (CKD-EPI$_{2009}$)** | **CKD G3-G5 with EKFC (not with CKD-EPI$_{2009}$)** |
|---|---|---|---|
| N | 29140 | 20846 | 8344 |
| eGFR EKFC (ml/min/1.73 m$^2$) | 50.1 (41.3-55.7) | 45.7 (37.5-51.1) | 57.5 (55.8-58.8) |
| eGFR CKD-EPI$_{2009}$ (ml/min/1.73 m$^2$) | 54.9 (45.3-60.8) | 50.1 (41.0-55.9) | 63.0 (61.5-65.0) |
| N with serum potassium values | 27484 | 19784 | 7747 |
| Serum potassium (mmol/L) | 4.60 (4.30-4.90) | 4.60 (4.30-4.90) | 4.50 (4.30-4.80) |
| Prevalence hyperkalemia, n (%) | 5117 (18.6%) | 4056 (20.5%) | 1061 (13.8%) |

Table S3. Prevalence of hyperkaliemia according to diabetes status.

| Hyperkaliemia | No diabetes | Diabetes |
|---|---|---|
| 16.9% vs 9.7%, No | 138462 (90.3%) | 16004 (83.1%) |
| Mild | 13569 (8.8%) | 2761 (14.3%) |
| Moderate | 1285 (0.8%) | 444 (2.3%) |
| Severe | 101 (0.066%) | 57 (0.30%) |

The diabetes status was unknown in 21,591 participants

Table S4. Prevalence of hyperkaliemia in people without diabetes according to eGFR G category.

| No diabetes | Hyperkaliemia | | | |
|---|---|---|---|---|
| G Category | No | Mild | Moderate | Severe |
| G1 | 50481 (93.6%) | 3229 (6%) | 202 (0.4%) | 7 (<0.1%) |
| G2 | 71054 (89.8%) | 7395 (9.3%) | 653 (0.8%) | 32 (<0.1%) |
| G3 | 15785 (84.3%) | 2604 (13.9%) | 309 (1.7%) | 20 (0.1%) |
| G4 | 882 (71.0%) | 267 (21.5%) | 83 (6.7%) | 11 (0.9%) |
| G5 | 142 (51.3%) | 66 (23.8%) | 38 (13.7%) | 31 (11.2%) |

Table S5. Prevalence of hyperkaliemia in people with diabetes according to eGFR G category.

| Diabetes | Hyperkaliemia | | | |
|---|---|---|---|---|
| G Category | No | Mild | Moderate | Severe |
| G1 | 2932 (90.7%) | 270 (8.4%) | 31 (1%) | 0 (0%) |
| G2 | 8493 (85%) | 1319 (13.2%) | 172 (1.7%) | 11 (0.1%) |
| G3 | 3989 (77.2%) | 985 (19.1%) | 175 (3.4%) | 21 (0.4%) |
| G4 | 495 (70.0%) | 154 (21.8%) | 47 (6.6%) | 11 (1.6%) |
| G5 | 78 (54.5%) | 32 (22.4%) | 19 (13.3) | 14 (9.8%) |

Table S6. Correlation matrix for predictive variables in the logistic multivariate model and VIF values.

| | eGFR | Platelets | Urea | MCV | HbA1C | Age | Hb | Calcium | Phosphate |
|---|---|---|---|---|---|---|---|---|---|
| eGFR | 1.00 | 0.19 | -0.48 | -0.19 | -0.17 | -0.75 | 0.09 | -0.08 | 0.09 |
| Platelets | 0.19 | 1.00 | -0.11 | -0.15 | 0.00 | -0.19 | -0.11 | 0.07 | 0.13 |
| Urea | -0.48 | -0.11 | 1.00 | 0.09 | 0.13 | 0.30 | -0.15 | 0.00 | 0.06 |
| MCV | -0.19 | -0.15 | 0.09 | 1.00 | -0.06 | 0.23 | 0.10 | 0.01 | -0.04 |
| HbA1C | -0.17 | 0.00 | 0.13 | -0.06 | 1.00 | 0.20 | -0.01 | 0.05 | 0.00 |
| Age | -0.75 | -0.19 | 0.30 | 0.23 | 0.20 | 1.00 | -0.11 | 0.02 | -0.18 |
| Hb | 0.09 | -0.11 | -0.15 | 0.10 | -0.01 | -0.11 | 1.00 | 0.20 | -0.12 |
| Calcium | -0.08 | 0.07 | 0.00 | 0.01 | 0.05 | 0.02 | 0.20 | 1.00 | 0.04 |
| Phosphate | 0.09 | 0.13 | 0.06 | -0.04 | 0.00 | -0.18 | -0.12 | 0.04 | 1.00 |

VIF values

| eGFR | Platelets | Urea | MCV | HbA1C | Age | Hb | Calcium | Phosphate |
|---|---|---|---|---|---|---|---|---|
| 2.965 | 1.116 | 1.605 | 1.092 | 1.064 | 2.422 | 1.206 | 1.053 | 1.110 |
